# Persistence of subclinical deformed wing virus infections in honeybees following *Varroa* mite removal and a bee population turnover

**DOI:** 10.1371/journal.pone.0180910

**Published:** 2017-07-07

**Authors:** Barbara Locke, Emilia Semberg, Eva Forsgren, Joachim R. de Miranda

**Affiliations:** Department of Ecology, Swedish University of Agricultural Sciences, Uppsala, Sweden; University of Salford, UNITED KINGDOM

## Abstract

Deformed wing virus (DWV) is a lethal virus of honeybees (*Apis mellifera*) implicated in elevated colony mortality rates worldwide and facilitated through vector transmission by the ectoparasitic mite *Varroa destructor*. Clinical, symptomatic DWV infections are almost exclusively associated with high virus titres during pupal development, usually acquired through feeding by *Varroa* mites when reproducing on bee pupae. Control of the mite population, generally through acaricide treatment, is essential for breaking the DWV epidemic and minimizing colony losses. In this study, we evaluated the effectiveness of remedial mite control on clearing DWV from a colony. DWV titres in adult bees and pupae were monitored at 2 week intervals through summer and autumn in acaricide-treated and untreated colonies. The DWV titres in Apistan treated colonies was reduced 1000-fold relative to untreated colonies, which coincided with both the removal of mites and also a turnover of the bee population in the colony. This adult bee population turnover is probably more critical than previously realized for effective clearing of DWV infections. After this initial reduction, subclinical DWV titres persisted and even increased again gradually during autumn, demonstrating that alternative non-*Varroa* transmission routes can maintain the DWV titres at significant subclinical levels even after mite removal. The implications of these results for practical recommendations to mitigate deleterious subclinical DWV infections and improving honeybee health management are discussed.

## Introduction

Deformed wing virus (DWV) is a prevalent single-stranded RNA virus of affecting the European honeybee (*Apis mellifera*). At highly elevated titres, it causes wing deformities in developing pupae, resulting in flightless adults that die shortly after emerging [1], [2], [3]. Expression of the characteristic symptoms is however largely dependent on how the bee acquires the DWV infection [4]. Typically, in its natural state, this virus occurs in honeybee colonies as an asymptomatic covert infection that is maintained in the colony through horizontal transmission pathways between bees such as trophallaxis, cannibalism, cleaning and salivary gland secretions [2], [5] and through vertical transmission from infected parents to their progeny [6], [7], [8]. The epidemiology of DWV has however been dramatically altered by the introduction of the invasive honeybee ectoparasitic mite, *Varroa destructor* [9]. This mite feeds on the hemolymph of developing pupae and adult bees, acting as a highly efficient vector of DWV by injecting the virus particles directly into bee hemolymph [10]. The *Varroa* mite also activates latent DWV infections through host immunosuppression, indirectly stimulating virus replication in bees [11]. These features of mite-vectored transmission lead to increased infection levels within individual parasitized bees [4]. Morphological DWV symptoms typically occur at high infection levels (> 10 ^10^ genome copies / bee) that are almost exclusively coupled with acquiring the virus during the pupal stage via the feeding behaviour of the ectoparasitic mite [2], [4]. High mite infestations within a honeybee colony ultimately lead to an overt DWV epidemic causing colony death within a few years [12], [13].

DWV is now considered a global epidemic driven by the world-wide spread of the *Varroa* mite [14] and has been implicated in the high rate of honeybee colony losses experienced in Europe and the US [9], [15], [16], [17], [18]. Accordingly, the *Varroa* mite is considered a severely damaging pest to the European honeybee due to its role as a virus vector and consequently the most significant economic threat to apiculture on a global scale [14], [19]. In order to prevent colony losses caused by the *Varroa* mite—virus infection epidemic, beekeepers must reduce or limit the growth of the mite population within the colony to break the vector transmission route of DWV. This is often done by using in-hive pesticides (acaricides) that specifically target the *Varroa* mite such as a tau-fluvalinate acaricide [20]. However, DWV can potentially induce colony losses independent of *Varroa* mite infestation even after mites are removed [21].

The aim of this study was to quantify the DWV infection dynamics during and following a *Varroa* mite removal treatment to evaluate the time necessary to clear a DWV infection from a honeybee colony after mites are removed. Such information would be valuable for improving honeybee health management and colony survival by optimizing the duration and the timing of acaricide treatments.

## Methods

### Experimental design and sampling

Six honeybee colonies, that had survived the 2013/2014 winter with relatively high mite infestations and with a large proportion of adult bees showing symptomatic DWV infections, were split and re-queened in the spring of 2014 to give a total of 12 (six pairs) experimental colonies. The colonies were tested in Uppsala, Sweden (Ultuna: N 59° 49'4,31 E 17° 39'24,60) and originate from the local bee population. Each pair of split colonies was equalized for brood amounts and adult bees. One colony from each pair was assigned to be treated with Apistan^™^ (tau-fluvalinate) according to manufacturer recommendations while the other colony received no mite-control treatment. Apistan^™^ is a potent synthetic pyrethroid acaricide with up to 98–100% efficacy against Varroa mite infestation [20]. The Apistan^™^ strips remained in the treated colonies for the entire study in order to sustain a low mite infestation since there was a high risk of mite re-invasion from drifting bees from the untreated mite-infested colonies in the same apiary.

Samples of 100–200 adult bees and 10 uninfested pupae were collected from each of the 12 colonies in two week increments starting two weeks before the Apistan was applied to the treated colonies. The last sample was 14 weeks after the treatment application. This sampling strategy produced a total of 9 sample time points between the 12^th^ of June and the 2^nd^ of October 2014. This time interval spans both the regular summer turnover of the adult bee populations and the production of the long-lived winter bees, which in Sweden occurs in late August [22]. No pupae were sampled on the last sampling date because the colonies were not rearing brood this late in the season. All experimental colonies overwintered and were examined in the spring on the 3^rd^ of March for overwintering survival.

The mite infestation rates in the colonies were determined by washing the adult bee samples in soapy water to dislodge any mites, and collecting these on a fine sieve [23]. These mites were counted and measured as a proportion of the number of adult bees in the sample [23]. Thirty adult bees from each of these samples were delegated for quantitative molecular analysis of DWV infection.

### RNA purification

The adult bee samples were extracted as a bulk sample of 30 bees and the pupae samples were extracted as a bulk sample of 10 pupae. The samples were placed in plastic mesh bags and ground to powder using liquid nitrogen and a pestle. To each ground sample, 200 μL per bee of GITC-buffer [24] containing 1% β-Mercaptoethanol was added, followed by shaking, to produce a primary homogenate. Total RNA was extracted from 100 μL of this homogenate by a QiaCube robot following the RNeasy protocol for plants (Qiagen). The RNA was eluted in 50 μL RNase-free water, the RNA concentration was estimated by NanoDrop and the sample was stored at -80°C until further use.

### RT-qPCR

The amount of deformed wing virus (DWV) RNA and RP49 mRNA (an internal reference gene for normalizing between-sample differences in RNA quantity and quality) in the adult and pupal bee samples was determined using reverse transcription quantitative PCR (RT-qPCR), using the iScript One Step RT-PCR kit (Bio-Rad) with SYBR Green as the detection chemistry and the Bio-Rad CFX connect thermocycler. Before RT-qPCR the RNA samples were diluted to a uniform concentration of 20 ng/μL to avoid concentration-dependent effects on RT-qPCR efficiency [24]. The reactions were performed in a 20 μL volumes containing 0.2 μM each of the forward and the reverse primer (S1 Table), 3 μL RNA, 10 μL SYBR Green RTmix and 0.4 μL of iScript reverse transcriptase. The thermocycling profile for both assays was as follows: 10 min at 50°C for cDNA synthesis, 5 min at 95°C for inactivation of the reverse transcriptase following 40 cycles of 10 sec. at 95°C for denaturation and 30 sec. at 58°C for annealing/extension and data collection. This profile was followed immediately by a Melting Curve analysis to confirm the identity of the amplification products, by incubating for 10 sec. at 95°C followed by reading the fluorescence at 0.5°C with increments from 65°C to 95°C. For both assays, a 10-fold serial dilution series of a positive cloned (plasmid) control of known concentration was also run on each reaction plate, as well as negative (water) controls, to establish the calibration curves for absolute quantification, as performed by the BioRad CFX software. The RT-qPCR data were subsequently converted to estimated copy numbers of each target RNA per bee as described previously [25].

### Statistical analysis

The DWV data for both adult bees and pupae were log transformed to meet assumptions of normally distributed data for parametric analysis. A linear maximum likelihood repeated-measures model (SAS, proc MIXED) was used to analyze the effects of acaricide treatment, time and a treatment*time interaction on the DWV titres in adult bees and pupae and the adult bee *Varroa* mite infestation rate. The *Varroa* mite infestation rate was included as an independent explanatory variable in the model. The covariance structure for the repeated factor was selected based on the Aikaike’s information criteria [26]. The assumption of normality and equal variance was verified by analysis of residuals [26].

## Results

**Fig 1:**
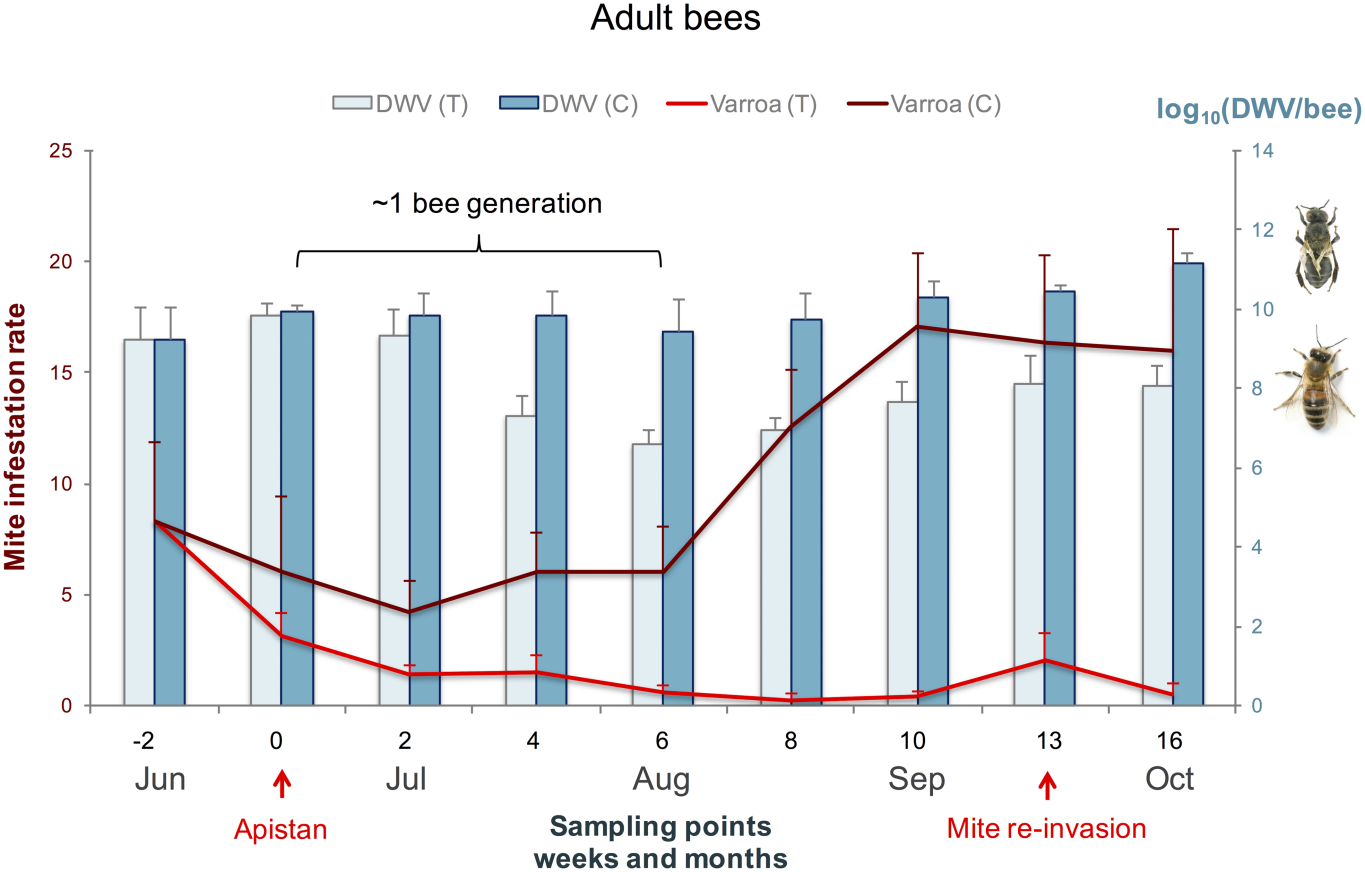
Average DWV titres in adult bees. The average DWV titres with standard error bars in adult bees in Apistan treated colonies (light bars) and untreated control colonies (dark bars) are presented on the right y-axis. The symptomatic bee with deformed wings and the asymptomatic bee represent the difference in titre amounts between clinical and sub-clinical DWV on the right y-axis. The average mite infestation rates with standard error bars in treated colonies (light red lines) and untreated colonies (dark red lines) are presented on the left y-axis. The sampling points are presented progressively in weeks (and months) in relation to when the Apistan treatment was administered.

The Apistan treatment effectively reduced the *Varroa* mite population of the treated colonies within 6 weeks after the Apistan was applied (Fig 1, Table 1). The mites were not completely eliminated from the treated colonies but this was most likely an artifact of mite-re-invasion from the neighbouring untreated colonies in the same apiary (Fig 1). During this 6-week period, the adult DWV titres in the treated colonies were reduced 1000-fold relative to those of the untreated colonies, a differential that was maintained to the end of the season (Fig 1). This 6-week period equates to the average life-span of a summer bee and therefore represents a full demographic turnover of the adult bee population. However, the adult DWV titres of both the treated and untreated colonies increased about 10-fold from its lowest point in mid-August to the final sampling in mid-October. This period coincides with the production of long-lived winter bees, and therefore represents a different demographic phase. Statistical analyses confirmed that the treatment had a significant effect on adult DWV titres throughout the study period, and that a significant part of this effect could be explained by the covariation in mite infestation rates (Table 1). This explanatory effect of the mite infestation rates would be largely from the first phase, where both infestation rates and DWV titres decline in the treated colonies.

**Table 1:** Results of the maximum likelihood repeated measures model analysis.

*Response variables*	DWV in Adult bees		DWV in pupae		Mite infestation rate	
	*F*	*P*	*F*	*P*	*F*	*P*
***Explanatory variables***						
Treatment	*F*_1,10_ = 12.03	**0.0060**	*F*_1,10_ = 0.44	0.5234	*F*_1,10_ = 26.60	**0.0005**
Date	*F*_8,78_ = 3.61	**0.0013**	*F*_7,61_ = 1.04	0.4157	*F*_8,79_ = 1.41	0.2053
Treatment * Date	*F*_8,78_ = 1.55	0.1527	*F*_7,61_ = 1.06	0.4020	*F*_8,79_ = 2.92	**0.0066**
Mites infestation rate	*F*_1,78_ = 12.00	**0.0009**	*F*_7,61_ = 8.69	**0.0045**	-	-

**Fig 2:**
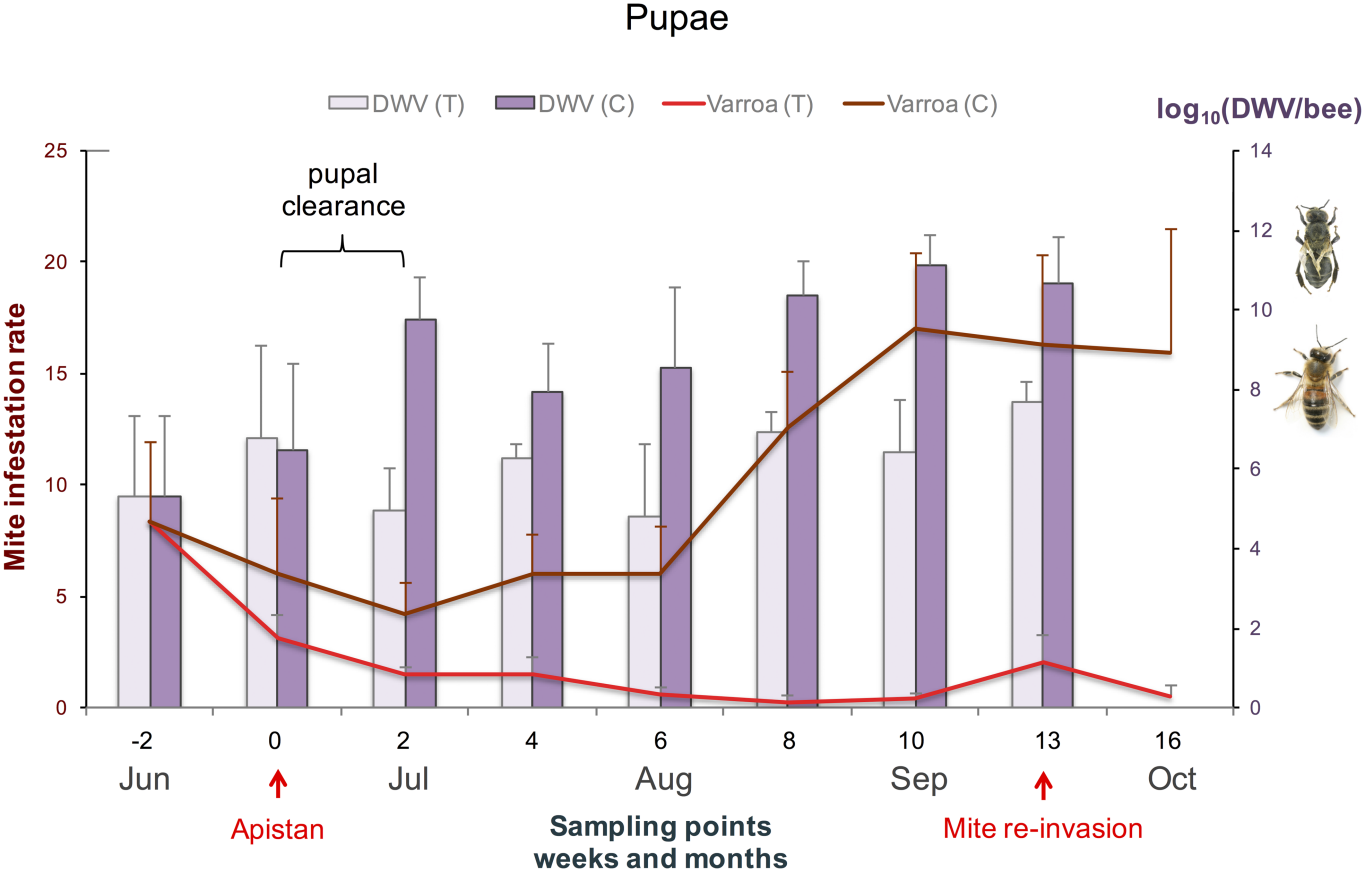
Average DWV titres in pupae. The average DWV titres with standard error bars in pupae in Apistan treated colonies (light bars) and untreated control colonies (dark bars) are presented on the right y-axis. The symptomatic bee with deformed wings and the asymptomatic bee represent the difference in titre amounts between clinical and sub-clinical DWV on the right y-axis. The average mite infestation rates with standard error bars in treated colonies (light red lines) and untreated colonies (dark red lines) are presented on the left y-axis. The sampling points are presented progressively in weeks (and months) in relation to when the Apistan treatment was administered.

Most of the remaining treatment effect comes from the second phase, where the DWV titre differences between treated and untreated colonies are maintained despite the increase in infestation rate in the untreated colonies. These trends for the adult DWV titres were largely mirrored by those for the pupae (Fig 2), but with greater variability, both between colonies at each time-point and between time-points. While the effect of treatment on adult bee samples was mostly reflected in a reduction in DWV titres in the treated colonies, the treatment differential on pupal samples was mostly reflected in a faster increase in DWV titres in untreated colonies. Since this coincides with the increase of the adult bee mite infestation rate in the untreated colonies, there was also a very strong explanatory effect of mite infestation rate on pupal DWV titres (Table 1). Removing the adult bee mite infestation rate as an explanatory variable for pupal DWV titres shifted the significance to the main treatment effect, which became marginally significant as a result (*F*_1, 10_ = 6.26; *P* = 0.0314). This indicates that the effect of the Apistan treatment on DWV titres in pupae is almost entirely due to its effect on the colony mite infestation rate, rather than directly on the virus infection. Nevertheless, despite the mite removal, the average pupal DWV titres in treated colonies were higher in the autumn than they were at any other time during the study, over 10^7^ genome copies / bee (Fig 2), attesting to the potency of the alternative transmission routes in maintaining the epidemic’s momentum. On the last sampling date of our study it was not possible to collect pupal samples since the colonies were no longer rearing new bees and would remain so through the winter.

The following spring the experimental colonies were checked for winter survival. One of the six Apistan treated colonies died during the autumn, before the final sampling, due to insufficient brood rearing. Three of the six untreated control colonies died during the winter (50% survival) These moribund colonies had the 2^nd^, 3^rd^ and 4^th^ highest adult DWV titres as well as the 1^st^, 2^nd^ and 6^th^ highest mite infestation rates of all colonies at the final sampling point the previous autumn.

## Discussion

The broad pattern of DWV dynamics in the experimental colonies following mid-summer acaricide treatment can be divided into two phases. The first phase is characterized by a drastic reduction in DWV titres, coinciding with the removal of *Varroa* from the colonies and reaching its maximum effect 6 weeks after the start of treatment. This is followed by the second phase where the DWV titres increase again slightly, with a parallel increase observed in the untreated colonies. The pattern is clearer for the adult bees, where the main effect is a reduction of DWV titres in the treated colonies, than for the pupae, where the main effect is a faster increase in DWV titres for the untreated colonies.

The reduction of adult DWV titres in the treated colonies of this study is consistent with the strong influence *Varroa* vectored transmission has on increasing the DWV titres in honeybee colonies [2], [4], [5], [10] [13], [27], [28]. The extent of the reduction (about 1000-fold) takes the adult DWV titres from clinical (>10^11^ copies DWV/bee) to subclinical levels (<10^8^ copies DWV/bee), which is sufficient to ensure winter survival. Clinical DWV symptoms in naturally infected bees typically start to appear at >10^10^ copies DWV / bee, although there is considerable overlap between symptomatic and asymptomatic bees [29].

Although the DWV titres at the final sampling of the winter bees in this study were low enough to avoid the most damaging symptomatic effects, they were still high enough to be relevant to bee health and performance. Sub-clinical DWV infections often have sub-lethal effects, such as reduced life span [30], flight performance [31], foraging age and efficiency [32]. The persistence of high sub-lethal DWV titres in the treated colonies, well after all the mites were removed from the colonies, shows the importance of alternative DWV transmission routes (most likely oral) in sustaining the momentum of the DWV epidemic in the absence of mite-mediated transmission. Doses of around 10^7^ virus particles/bee are usually sufficient for successful oral infection of larvae or adult bees [33]. The more unexpected result was the progressive increase of subclinical DWV titres in the treated colonies during the second phase of the experiment in both the pupal and adult samples, well after the mites were nearly completely removed from these treated colonies, instead of continuing to decrease or even level off. There are several possible factors that could have influenced this increase. The slight mite re-invasion towards the end of the study, likely from the nearby untreated colonies [34], was probably not strong enough and too late in the season to explain the 10-fold increase in DWV titres and occurs well after the upward trends were established. Drifting bees from untreated colonies can likewise be excluded as an influencing factor since it cannot explain the increase in pupal DWV titres. Regular bee turnover is also unlikely to be a major factor as the bees produced during the first phase are progressively less DWV-infected, while the pupae and the adults developing from the larvae feed by the first phase bees are progressively more infected. We suspect that this progressive increase in DWV titres from mid-August onwards is related to the nature of the production of the winter bees [35], with their unique physiological and functional traits [36]. Young nurse bees consume pollen and convert the nutrients to fats and the life-extending storage protein vitellogenin in their fat bodies [37]. These fat bodies are also major replication sites for DWV and similar viruses [38], [39]. During brood-rearing, the nutrients and constituents (and virus) produced in the fat bodies are used up to produce a proteinaceous secretion for feeding (and infecting) young larvae [40]. As brood-rearing slows down in autumn, more of the fat body resources (and virus) is retained as the bee prepares for surviving a long period of foraging dearth and becomes a ‘winter’ bee [41], [42]. Simultaneously, the collective nursing activity becomes increasingly focused on a shrinking population of (winter bee) larvae. These larvae are thus increasingly likely to receive a full infectious dose of DWV from infected nurse bees, relative to periods of high brood-rearing activity. Surges of Brood-rearing may thus also help explain the variability in pupal DWV titres during the foraging season, and between colonies. A third possible factor for the late season progressive increase of DWV titres could be the extended exposure of the treated colonies to tau-fluvalinate, which was previously shown to be associated with a temporary increase in DWV titres in late-season pupae and adult bees [25].

Honeybee colony death most often occurs during the winter months in temporal climates during the sensitive overwintering phase of the annual colony cycle [36]. The long-lived overwintering bees (surviving > 200 days) are produced late in the summer or autumn and will be responsible for foraging and rearing the next generation for the colony in the following spring. Due to this static population structure, where there is no adult populations turnover for several months, the health status of these long-lived winter bees is therefore of particularly critical importance for successful overwintering and colony survival. If *Varroa* control treatments are administered too late in the season, the overwintering bees will have already been reared under *Varroa*-infested conditions and may be too ill-affected by virus infections to survive the winter, even if the mite treatment itself was effective at removing the mites.

The reduction of DWV titres in the treated colonies from clinical to subclinical levels not only paralleled the removal of mites but also, and perhaps more importantly, occurred during a turnover of the adult bee population (1 bee generation is approximately 38 days or nearly 6 weeks). The turnover of the adult bee population in a remedial *Varroa* treatment regime is probably more critical than previously realized. Highly infected adult bees must be replaced with a new generation of adults reared in a *Varroa*–free environment, so that new and progressively healthier bees will nurse and feed the larvae of the long-lived overwintering bees. By conducting our study over the summer months it was possible to observe the influence of the bee population dynamics in addition to mite removal on DWV infections. A previous study using Apistan treatment in the late summer showed high DWV titres in adult bees (> 10^10^ copies / bee) and pupae (> 10^9^ copies / bee) over the entire 6-week study [25]. Martin *et al*. [43] surveyed bees over the winter and using serological detection found a faster reduction of DWV when colony mite removal was performed in the summer rather than autumn.

The DWV titres in the adult winter bees is dependent on what happens previously in the pupae [2]. In this experiment, the progressively increasing DWV titres in untreated colonies from early August onwards, when the brood rearing slows down, corresponds to the period when the mite infestation rate also increases [13]. This consequently increases the proportion of winter-bee pupae with clinical DWV titres and compromises the colony’s chance of winter survival. For those pupal DWV titres to remain below 10^10^ copies DWV/bee, treatment should not be later than at least 6 weeks before the end of brood rearing to allow for a generation turnover within the bee population.

Our data demonstrates the importance of the alternative, non-Varroa virus transmission routes (e.g. oral transmission) for maintaining DWV titres within the colony at significant subclinical levels, even after the *Varroa* mites have been removed. Remedial treatment of honeybee colonies with high mite infestations and consequent high DWV titres may save the colony from immediate winter death, but subclinical DWV levels in overwintering bees could result in an insufficient number of bees for colony growth in the spring [13], [16], [32], causing an increased risk for damage by the *Varroa*–driven DWV epidemic the following year. To mitigate subclinical effects of DWV, we extend the above practical recommendation to continuously monitor and maintain low mite infestation rates in colonies by incorporating other integrated pest management (IPM) strategies to avoid irreversibly high DWV levels from building up, rather than relying on late season remedial treatment alone.

## Supporting information

S1 File Dataset. Persistence of subclinical deformed wing virus infections in honeybees following *Varroa* mite removal and a bee population turnover.
(XLSX)

S1 Table Primer sequences and performance indicators for the RT-qPCR assays. Primer sequences and performance indicators, including the melting temperature of PCR products, for the RT-qPCR assays for DWV and internal reference gene RP-49.
(PDF)
