# Supplementary material for: Persistence of subclinical deformed wing virus infections in honeybees following Varroa mite removal and a bee population turnover
Source: PLoS One. 2017 Jul 7;12(7):e0180910. doi: 10.1371/journal.pone.0180910 (PMC5501613; doi:10.1371/journal.pone.0180910)
Supplement: S1 Table — Primer sequences and performance indicators, including the melting temperature of PCR products, for the RT-qPCR assays for DWV and internal reference gene RP-49. (PDF) [file pone.0180910.s002.pdf]

S1 Table. Primer sequences and performance indicators for the RT-qPCR assays. Primer sequences and performance indicators, including the melting temperature of PCR products, for the RT-qPCR assays for DWV and internal reference gene RP-49.

Assay	Primers	Sequence (5'-3')	T_m	Size	E	R^2
DWV	DWV-F1425	CGTCGGCCTATCAAAG	51.8	417bp	71.7	1
	DWV-B1806	CTTTTCTAATTCAACTTCACC	52.0			
RP-49	RP49-qF	AAGTTCATTCGTCACCAGAG	55.3	205bp	93.1	0.999
	RP49-qB	CTTCCAGTTCCTTGACATTATG	56.5			
